# CARDIOSIM^©^: The First Italian Software Platform for Simulation of the Cardiovascular System and Mechanical Circulatory and Ventilatory Support

**DOI:** 10.3390/bioengineering9080383

**Published:** 2022-08-11

**Authors:** Beatrice De Lazzari, Roberto Badagliacca, Domenico Filomena, Silvia Papa, Carmine Dario Vizza, Massimo Capoccia, Claudio De Lazzari

**Affiliations:** 1 Department of Human Movement and Sport Sciences, “Foro Italico” 4th University of Rome, 00135 Rome, Italy; 2 Department of Clinical, Internal Anesthesiology and Cardiovascular Sciences, “Sapienza” University of Rome, 00185 Rome, Italy; 3 Department of Cardiac Surgery, Leeds General Infirmary, Leeds Teaching Hospitals NHS Trust, Leeds LS1 3EX, UK; 4 Department of Biomedical Engineering, University of Strathclyde, Glasgow G4 0NW, UK; 5 National Research Council, Institute of Clinical Physiology (IFC-CNR), 00185 Rome, Italy; 6 Faculty of Medicine, Teaching University Geomedi, Tbilisi 0114, Georgia

**Keywords:** CARDIOSIM^©^, numerical simulator, lumped-parameter model, e-learning, mechanical circulatory support, ventilatory, cardiovascular system, heart failure, clinician

## Abstract

This review is devoted to presenting the history of the CARDIOSIM^©^ software simulator platform, which was developed in Italy to simulate the human cardiovascular and respiratory systems. The first version of CARDIOSIM^©^ was developed at the Institute of Biomedical Technologies of the National Research Council in Rome. The first platform version published in 1991 ran on a PC with a disk operating system (MS-DOS) and was developed using the Turbo Basic language. The latest version runs on PC with Microsoft Windows 10 operating system; it is implemented in Visual Basic and C++ languages. The platform has a modular structure consisting of seven different general sections, which can be assembled to reproduce the most important pathophysiological conditions. One or more zero-dimensional (0-D) modules have been implemented in the platform for each section. The different modules can be assembled to reproduce part or the whole circulation according to Starling’s law of the heart. Different mechanical ventilatory and circulatory devices have been implemented in the platform, including thoracic artificial lungs, ECMO, IABPs, pulsatile and continuous right and left ventricular assist devices, biventricular pacemakers and biventricular assist devices. CARDIOSIM^©^ is used in clinical and educational environments.

## 1. Introduction

Cardiovascular modeling has significantly increased our knowledge of physiological mechanisms. Simplified representations of complex biological systems can be used to study their behavior at different levels [1]. The Cardiac Physiome project is the most ambitious and successful application of mathematical and computational modeling at the present time. The aim is the advancement of our understanding of physiology using a quantitative multi-scale approach.

Although still far from clinical application on a daily basis [2,3], patient-specific modeling has consistently shown significant potential [4,5,6]. The modeling approach should be robust, realistic enough and easy to understand to gain wide acceptance. The ability to deliver reliable results within the time and constraints of clinical practice is the key element. The clinician remains the ultimate decision-maker.

Models based on the pressure–volume relationship and lumped-parameter description of the cardiovascular system may be a suitable choice given their simplicity and versatility [7,8,9,10,11,12,13]. Although they provide less detailed predictions of pressure and flow waveforms, these models have shown great flexibility in simulating the hemodynamics of different cardiovascular conditions and therapeutic interventions with the potential to be run in real time on any computer device [5]. The successful clinical application of this approach requires further training of the medical community and a different mindset to appreciate its potential [13,14,15].

A simulation approach based on patient-specific modeling may be used as an additional tool to guide therapeutic intervention in advanced heart failure, aimed at treatment optimization and possibly outcome prediction [16,17]. More specifically, the analysis of the potential effects of a left ventricular assist device (LVAD) on heart failure patients may help decide about treatment suitability in terms of which patient may benefit the most. The native ventricular behavior can be modeled according to the time-varying elastance theory [18,19,20], which remains a landmark despite its limitations [21] and previous criticism when applied to a mechanically supported left ventricle [22]. Significant elastance changes are observed during circulatory support with a pulsatile pump because of extreme and fast-changing loading conditions. Therefore, the relationship between elastance and contractility may be no longer applicable when a second pump is connected to the systemic circulation [22]. Elastance changes are also observed with continuous flow pumps. In fact, an increasing pump flow is related to a constant end-systolic volume, a decrease in end-diastolic volume and an elevated left ventricular pressure with a gradual increase in the slope (*Ees*) of the end-systolic pressure–volume relationship (ESPVR) to justify dissociation between contractility and elastance [22]. Although a linear model is sufficiently accurate [23,24,25] and adequate for realistic simulations of the instantaneous pressure–volume relation [26], recent multi-scale modeling of the cardiovascular system [27] based on previous approaches [28,29,30] has successfully addressed the limitations of the time-varying elastance theory, confirming load-dependence of ESPVR. Further modeling techniques can describe a failing cardiovascular system [31] and ventricular interactions [32,33,34]. To address the shortcomings of the original time-varying elastance theory, a nonlinear time-varying lumped-parameter model of the cardiovascular system [35] can be modified to include the interventricular septum and a rotor-dynamic, continuous flow LVAD [36]. In this context, the ESPVR is a unimodal function that takes into account the descending limb of the Frank–Starling curve, making it particularly suitable for studying ventricular interactions and the leftward septal shift following left ventricular decompression after LVAD insertion. 

The aim of this article is to give a bird’s eye view of the potential benefit of cardiovascular modeling and simulation in a clinical setting using CARDIOSIM^©^ (the first Italian software platform for simulation of the cardiovascular system and mechanical circulatory and ventilatory support), a long-standing and validated software developed by our group.

## 2. Materials and Methods

A narrative review was performed focusing on CARDIOSIM^©^ applications in cardiovascular simulation. A systematic approach was used for the study identification and selection. The article search was performed in Medline (through PubMed), Cochrane Library, Embase, Web of Science and Clinical Trials databases. A substantial literature search was conducted from 1991 to May 2022. Only articles published in the English language were considered. The MeSH keywords used were: “CARDIOSIM^©^”, “cardiovascular system”, “numerical simulation”, “mechanical assist device”, “mechanical ventilation”, “heart failure”, “ventricular assist device”, “time-varying elastance” and “software simulator”. The MeSH keywords were combined using the Boolean operators AND and NOT. The search was extended through the reference lists of the recruited texts. Relevant secondary references were also captured. 

**Figure 1:**
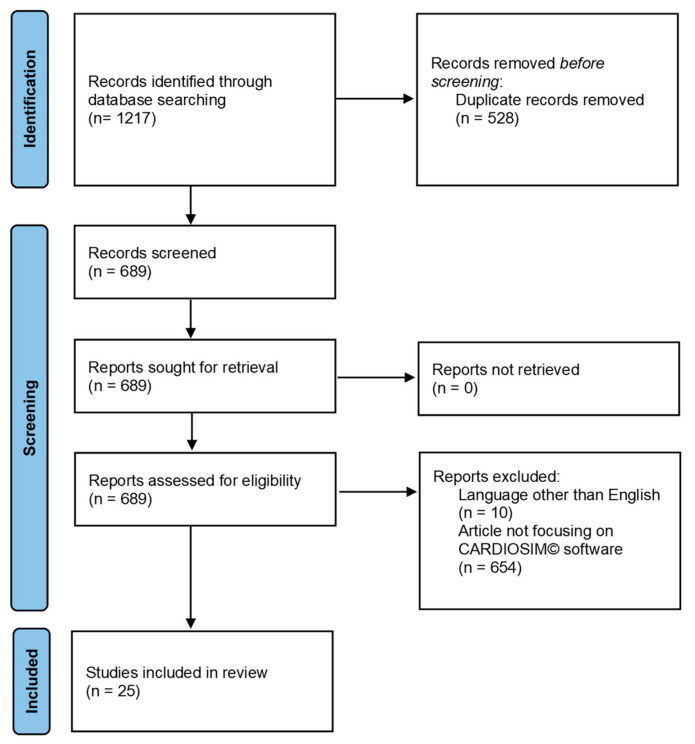
PRISMA flow chart obtained from the following link http://www.prisma-statement.org/documents/PRISMA_2020_flow_diagram_new_SRs_v1.docx (accessed on 29 May 2022).

Studies were considered eligible when investigating cardiovascular simulations using CARDIOSIM^©^ software. Exclusion criteria were: studies written in a language other than English; studies evaluating cardiovascular simulators different from CARDIOSIM^©^ software; reviews, metanalyses and commentaries. Two reviewers (BDL and MC) independently selected the articles by reading the titles and abstracts. Disagreements were resolved by discussion between the two reviewers or through third-party adjudication (CDL). After the selection, each title/abstract/full text was independently evaluated by each of the authors. A standardized approach was used to collect the data, which included the article’s first author, publication year, simulated condition, implemented simulator module and results. Figure 1 shows the PRISMA flow chart describing the number of papers recovered from each database, the number of documents excluded, etc.

### 2.1. Historical Overview

The first Italian software platform for simulation of the cardiovascular system and mechanical circulatory and ventilatory support was developed at the Institute of Biomedical Technology (ITBM of Rome) of the National Research Council (CNR) in 1991. In the previous two years, researchers of this institute and those of the National Heart, Lung, and Blood Institute (NHLBI) of the National Institutes of Health (NIH) laid the foundations for creating a software platform that implemented the numerical models of the cardiovascular system based on a modular approach to allow continuous updates and further development. The researchers from ITBM implemented the first version of the platform named CARDIOSIM^©^ using Turbo Basic language (released by Borland Software Corporation in 1987). The cardiovascular software simulator ran on an IBM-compatible PC with a disk operating system (MS-DOS) and with one megabyte of RAM.

**Figure 2:**
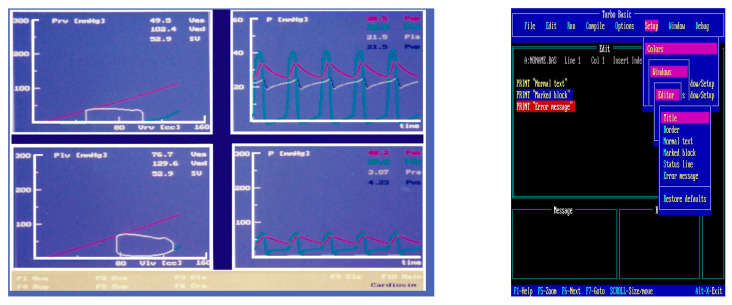
Screen output (**left**) from the first version of CARDIOSIM^©^ implemented using Turbo Basic language. Left lower (upper) window: left (**right**) ventricular pressure–volume loop with stroke volume (SV) and end-systolic (Ves) and end-diastolic (Ved) volume values. Right lower side of the screen output: instantaneous waveforms and mean values (calculated during a cardiac cycle) of systemic arterial (Pas) and right (Pra) atrial pressures. Right upper side of the screen output: instantaneous waveforms and mean values (calculated during a cardiac cycle) of pulmonary arterial (Pap) and left (Pla) atrial pressures. Right side: screenshot of the Turbo Basic language development environment.

The first version of the simulator was copyrighted in 1991 (copyright n. 320896, application date 22 July 1991) [37]. Figure 2 shows a screenshot from the first version of CARDIOSIM^©^ (left side) and an image of the Turbo Basic language development environment (right side).

Subsequently, a collaboration between researchers from the Polish Academy of Sciences and researchers from the Sapienza University of Rome led to the design and development of the second version of CARDIOSIM^©^. Visual Basic and C++ languages were implemented in the new version, which ran on a PC with Windows operating system. This version was protected by a second copyright in 1999 (copyright n. 001252, application date 15 May 1999) [37]. Over the years, new modules designed in collaboration with researchers from national and international institutions have been included within the platform. For example, one of the latest modules introduced within the platform can simulate the behavior of extra-corporeal membrane oxygenation (ECMO) support and the interaction with the cardiovascular network. This module was developed at the Cardiovascular Numerical/Hybrid Modelling Lab (Rome) of the Institute of Clinical Physiology (IFC-CNR) in collaboration with researchers from the Institute of Physiology of RWTH Aachen University (Germany); Department of Human Movement and Sport Sciences, “Foro Italico” 4th University of Rome; Department of Biomedical Engineering, University of Strathclyde (Glasgow); and Department of Clinical, Internal Anesthesiology and Cardiovascular Sciences, “Sapienza” University of Rome. Version 7.3.2 of the software platform is currently available. 

Over the years, the platform has been used as a research tool both in the bioengineering field and in the clinical environment, leading to studies that have been published in high-impact international journals.

In addition, CARDIOSIM^©^ has been used for the practice and learning needs of experienced professionals and those in training programs from different disciplines [8]. Simulation-based learning has become the way forward to develop the knowledge, skills and attitude of healthcare professionals whilst protecting patients from unnecessary risks. In medicine, simulation offers good scope for the training of interdisciplinary medical teams. An increasing number of national and international healthcare institutions and medical schools have benefited from the use of the CARDIOSIM^©^ platform. Many more are considering its use. In Italy, for example, the software simulator has been used to supplement the syllabus of a high-level master course for continuing medical and/or nursing education named “The Virtual Patient for Cardiology Training” [38] organized by “Sapienza” University. The course is run by a team of researchers from the IFC-CNR; the Institute of Physiology of RWTH Aachen University; and the Department of Clinical, Internal Anesthesiology and Cardiovascular Sciences, “Sapienza” University. The software has also been used to complement the “Models of Biological Systems” course for bioengineering students. To date, CARDIOSIM^©^ has been employed by six international institutions.

In 2011, a web page providing information about the platform and its potential applications, the various modules featured by the software and the references derived from the use of the CARDIOSIM© software simulator was created by the Biomedical Sciences Department of the CNR (DSB-CNR) [39].

### 2.2. Numerical Models of Ventricles, Atria and Septum

**Figure 3:**
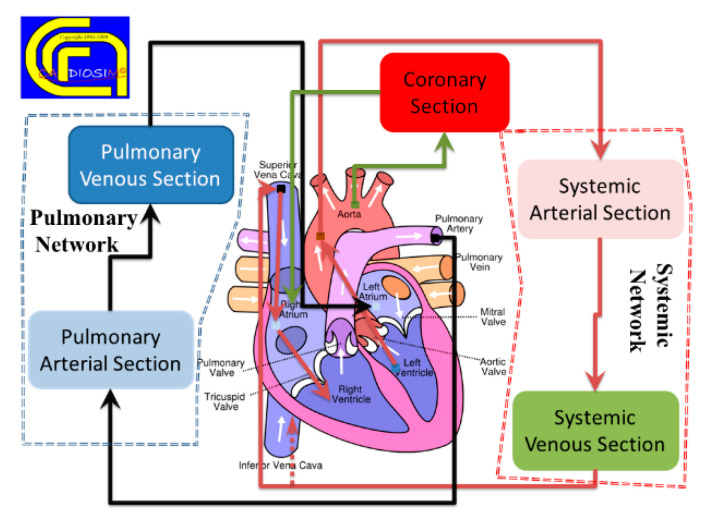
Schematic representation of the different sections implemented in the software cardiovascular simulator platform. (Reprinted with permission from [39], Copyright © 2022–2019 C. De Lazzari.)

CARDIOSIM^©^ software simulator platform has been developed to include seven different general sections of the whole circulation (Figure 3). Each section can be assembled using one of the different modules implemented using the zero-dimensional (0-D) representation. The complexity of the assembled model featuring the behavior of one of the seven sections depends on the context in which it must be studied.

The left (right) ventricular filling and ejection phases are described separately in the first version of the numerical simulator [10,40]. The time-varying elastance theory is used to reproduce the contraction and ejection phases [19,41,42]. This representation allows plotting the ventricular loops on the pressure–volume plane, with the end-systolic pressure–volume relationship (ESPVR) and the end-diastolic pressure–volume relationship (EDPVR). Therefore, Starling’s law of the heart can be reproduced. The behavior of the left (right) atrium is described as a linear capacity with constant compliance and unstressed volume neglecting the contractile atrial activity (Table 1).

In version 7.3.2 of the platform, a time-varying elastance model describes the behavior of both native ventricles and atria. The time-varying elastance theory is based on the instantaneous relationship between ventricular (atrial) pressure and volume. The mechanical properties and the different phases of each ventricle are synchronized with the ECG signal. The time-varying elastance was modeled as a function of both ventricular systolic and diastolic elastance. Finally, the ventricular activation function is modeled according to ECG timing. 

The left/right atrial time-varying elastance is modeled on the electro-mechanical interaction by synchronizing phases of the atrial cycle with the ECG signal. The left/right atrial time-varying elastance is a function of left/right atrial systolic elastance, left/right atrial diastolic elastance and left/right atrial activation function.

The concept of atrial/ventricular interdependence considers the properties of one atrium/ventricle to be a function of the properties of the contra-lateral one. The time-varying interatrial/interventricular septum is described as a function of interatrial/interventricular septum diastolic elastance, interatrial/interventricular septum systolic elastance and interatrial/interventricular septum systolic elastance [13,19,41,42,43]. 

### 2.3. Numerical Model of Systemic and Pulmonary Circulation

Systemic and pulmonary circulations are described with lumped-parameter models. Zero-dimensional models eliminate the variation in space and allow the description of pressure and flow as a function of time in a specific compartment of the circulatory system [44]. Zero-dimensional modeling is widely used in the study of the interaction between an assist device and the cardiovascular system [17,45,46,47] and for the analysis of average values of hemodynamic parameters.

Lumped-parameter modeling gives less detailed predictions of pressure and flow waveforms, but it has shown great potential and flexibility in clinical application with particular reference to the pathophysiology of heart failure, guidance for patient selection and the hemodynamic impact of device intervention [17,48]. A combined approach of 0-D modeling, pressure–volume analysis and modified time-varying elastance has a significant potential for daily use within the constraints of a clinical setting. 

Table 2 shows the evolution over time of numerical models of the systemic section using RLC elements.

### 2.4. Numerical Model of the Coronary Circulation

Models of the coronary circulation are implemented in CARDIOSIM^©^ assuming that local myocardial blood flow is governed by the waterfall mechanism. In the first version of the numerical software simulator, the behavior of the coronary bed was modeled with a resistor in series with a diode and a battery [10,40]. The diode–battery combination represents the intramyocardial pressure. The battery voltage is assumed proportional to left ventricular pressure (LVP), with a proportionality constant that decreases from lumen to epicardium where values are considered negligible. This electrical circuit cannot describe all the oscillatory pressure–flow relations observed experimentally. For instance, systolic arterial back-flow that occurs for low inflow pressures cannot be represented using the waterfall model.

Table 3 shows the evolution over time of numerical models of the coronary circulation.

### 2.5. Mechanical Ventilatory Assistance

Starting from the first version, CARDIOSIM^©^ has allowed the simulation of assisted mechanical ventilation by varying the mean value of intrathoracic pressure.

Any mode of artificial ventilation of the lungs (except continuous airway pressure) generates cycling changes in intrathoracic pressure. Different clinical studies have shown that the influence of mechanical ventilation support on hemodynamics in a steady state can be reproduced by changing the mean value of intrathoracic pressure [11,49,50]. The mean thoracic pressure (*Pt*) can be defined using the following equation:

$$P\left(t\right)=\frac{1}{T}\int_{0}^{T}p_{t}\left(t\right)dt$$

where *p_t_*(*t*) is the instantaneous thoracic pressure and *T* represents the ventilatory cycle time. Thus, by incorporating the thoracic pressure in all the numerical models of the different cardiovascular sections (i.e., ventricles, atria, thoracic aorta, pulmonary arterial section, etc.), it is possible to simulate the effects induced by mechanical ventilation simply by changing the level of *Pt*.

**Table 2:** Evolution over time of numerical models of the systemic section.

First Version	Second Version	Version 7.3.2
Systemic arterial section modeled with modified Windkessel (RLC) or three-cell model [10,40].	Systemic arterial section modeled with modified Windkessel (RLC) or three-ell model. (R is a resistance, L is an inertance and C is a compliance)	Systemic arterial section modeled with modified Windkessel (RLC) or three-cell model.
Systemic venous section modeled with RC elements.	Systemic venous section modeled with RC elements.	Systemic venous section modeled with RC elements.
--------	Systemic arterial module reproducing the behavior of both splanchnic and extra-splanchnic bed (both with 2-WM elements) and peripheral/venous circulation in active muscle compartment (using 2-WM elements).	Systemic arterial module reproducing the behavior of both splanchnic and extra-splanchnic bed (both with 2-WM elements) and peripheral/venous circulation in active muscle compartment (using 2-WM elements).
---------	---------	Systemic circulation modeled with: ascending aorta, carotid arteries, descending aorta, peripheral arteries, systemic veins circulation and vena cava section. The compartments are modeled with RC and RLC elements.
---------	---------	Systemic network modeled with: ascending, thoracic and abdominal aorta; superior (inferior) vena cava SVC (IVC); and lower and upper body [51]. The compartments are modeled with RC and RLC elements.

**Table 3:** Evolution over time of numerical models of the coronary section.

First Version	Second Version	Version 7.3.2
Waterfall model [6,7].	Waterfall model.	Waterfall model.
--------	RC model. The two resistances in series mimic the arteriolar, capillary and venous resistance. The capacitance mimics the large intramyocardial compliance.	RC model. The wo resistances in series mimic the arteriolar, capillary and venous resistance. The capacitance mimics the large intramyocardial compliance.
--------	--------	The coronary bed is composed of two main arteries (modeled with RC elements) perfusing the left and right ventricles.
--------	--------	RC model with subendocardial, middle and subepicardial layers of the left ventricular wall [52].

In addition, the software platform is suitable for carrying out various studies focused on the interaction between the cardiovascular system, mechanical ventilatory support and mechanical circulatory assist devices (MCADs) [12,51,52,53,54,55,56,57,58,59,60,61,62,63]. 

### 2.6. Mechanical Circulatory Assist Devices

Both pulsatile and continuous flow mechanical circulatory assist devices were implemented in the software platform. Left/right ventricular assist devices (LVADs/RVADs) can be connected either “in series” or in parallel mode. When the LVAD (RVAD) is connected “in series”, the pump draws blood from the atria and ejects it into the aorta (pulmonary artery). When the LVAD (RVAD) is connected in parallel mode, the pump draws blood from the ventricle and ejects it into the aorta (pulmonary artery). If necessary, both LVAD and RVAD can be activated simultaneously to obtain a BVAD configuration. 

A thoracic artificial lung (TAL) has also been implemented in CARDIOSIM^©^ using RLC elements. The numerical model was developed in cooperation with researchers from Innsbruck Medical University [62]. The connection to the pulmonary circulation can be either in series, in parallel or in a hybrid mode. The TAL input is connected to the right ventricular outflow tract in the hybrid mode. Outlet flow from the hybrid TAL splits between two grafts: the first outlet graft is connected to the pulmonary circulation through a resistance (R); the second one is linked to the left atrium through the RL element. These resistances allow blood flow splitting between the TAL and the pulmonary circulation. The hybrid mode enables sufficient levels of blood flow to both native lungs and TAL, while maintaining an intermediate load to the right ventricle [12]. 

The following extra-corporeal membrane oxygenation configurations were implemented in the software platform [51]:

Central veno-arterial ECMO (VA_RA-DA_-ECMO): ECMO draws blood from the right atrium (RA) and ejects it into the descending aorta (DA).
Veno-venous ECMO (VVI_VC-SVC_-ECMO): ECMO draws blood from the inferior vena cava (IVC) and ejects it into the superior vena cava (SVC).
Veno-arterial ECMO (VA_FV-TA_-ECMO): ECMO draws blood from the femoral vein (FV) and ejects it into the thoracic aorta (TA).

Table 4 shows the different numerical models of MCADs implemented within the software platform in the various versions. 

**Figure 4:**
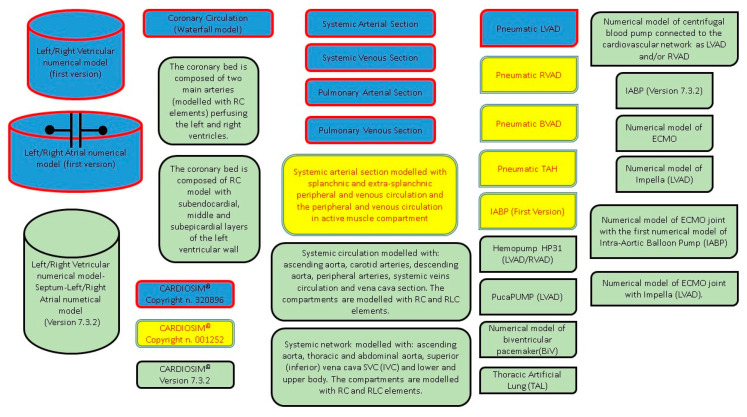
Graphical representation of all the modules implemented in CARDIOSIM^©^ software simulator. Blue modules were implemented in the first version (copyright n. 320896). Blue and yellow modules were implemented in the second version (copyright n. 001252). All modules (blue, yellow and green) are implemented in the latest version 7.3.2.

A graphical representation of the different modules implemented in the software platform can be observed in Figure 4.

### 2.7. Clinical Application of CARDIOSIM^©^

**Figure 5:**
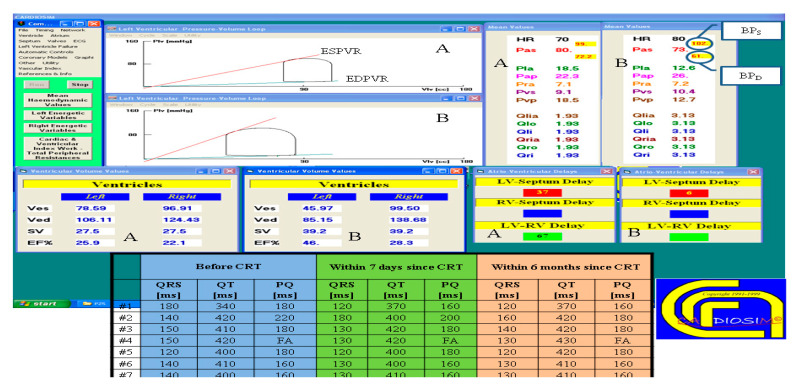
Screen output from CARDIOSIM^©^ reproducing the hemodynamic conditions of patient #5, simulated from hemodynamic parameters and ECG timing measured in the clinical environment, and represented on the left and right pressure–volume planes with the end-systolic (end-diastolic) pressure–volume relationship ESPVR (EDPVR). The mean (evaluated during the cardiac cycle) systolic arterial pressure (Pas), the mean pulmonary arterial pressure (Pap) and the input flow of the left (right) atrium Qlia (Qria) are listed in the screen output. The stroke volume (SV) with the left and right end-systolic (diastolic) ventricular volume Ves (Ved) are listed in the two tables on the left. “LV-Septum Delay” is the intraventricular delay time; “LV-RV Delay” is the interventricular delay time. The lower middle table shows the ECG timing parameters (PQ, QRS and QT duration) measured in seven patients before cardiac resynchronization therapy (CRT) and seven days and six months after CRT. (Reprinted with permission from [67], Copyright © 2022–2019 C. De Lazzari.)

Since the first version of CARDIOSIM^©^, the numerical simulator has been used to carry out studies both on animal models [64] and in a clinical setting [11,17,43,52,65,66]. Figure 5 shows a screen output from one of the latest versions of the software platform obtained during the analysis of hemodynamic parameters measured in a clinical setting. Seven patients underwent electrocardiographic and echocardiographic evaluation before and after biventricular pacemaker (BiV) implantation, more specifically 24 h, 7 days and 6 months following CRT. The effects of BiV were evaluated in a clinical setting. The cardiovascular condition of a patient before (A) and six months after (B) biventricular pacemaker implantation is derived [43].

**Figure 6:**
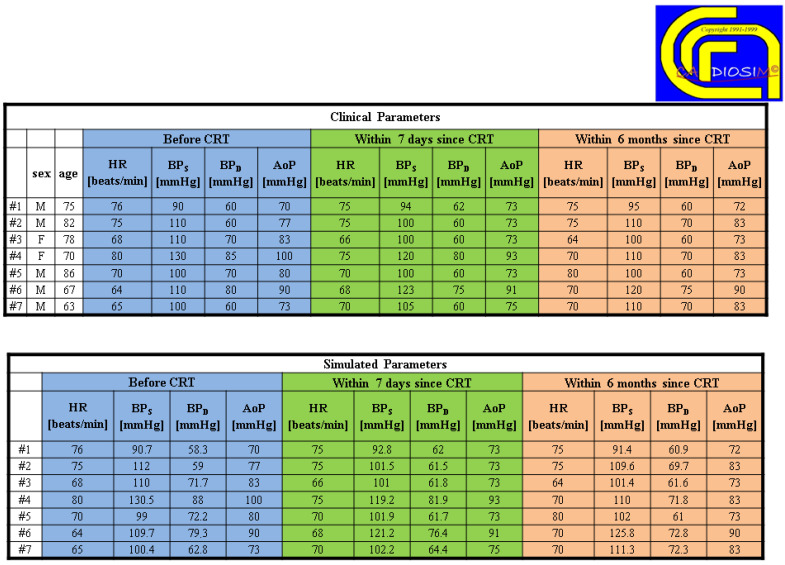
Comparison between clinical and simulated hemodynamic parameters (for seven different patients) before cardiac resynchronization therapy (CRT) and seven days and six months after CRT. (Reprinted with permission from [67], Copyright © 2022–2019 C. De Lazzari.)

Figure 6 shows two tables with clinical and simulated hemodynamic parameters before cardiac resynchronization therapy (CRT) and seven days and six months thereafter [43].

## 3. Discussion

Cardiovascular modeling with a view to clinical application based on a patient-specific approach may become a daily routine in the non-distant future. A model must be reliable and reproducible and reduce uncertainty to achieve its full potential [1,68]. While effective in the laboratory, almost all the decision support tools have failed when applied to clinical practice [69]. Therefore, it is essential to overcome skepticism by developing a strong, realistic model that can fulfill the expectations. The focus must be on modeling and simulation not as a substitute for clinical experience but as an additional tool to guide therapeutic intervention or predict clinical outcome: the clinician will be the ultimate decision maker. The development of a comprehensive and integrated description of the cardiovascular system based on lumped-parameter models, modified time-varying elastance and pressure–volume analysis of ventricular function is an attractive prospect with a view to clinical application. The differential equations can be solved relatively easily and yield answers in terms of pressure–volume loops and time-dependent tracings of pressure, flow and volume that may well help the decision process and management in the clinical setting. CARDIOSIM^©^ fulfills these requirements compared to other software such as CircAdapt Simulator, HeMoLab and Harvi. The CircAdapt Simulator is based on the CircAdapt model [70,71,72], which has been designed to simulate the dynamics of the heart and the circulation with the inclusion of a realistic relationship between pressure–volume load and tissue mechanics. The geometry of the components is obtained by adaptation to mechanical load. The implementation of the TriSeg model [73] enables realistic simulation of ventricular mechanics including interactions between left and right ventricles, dynamics of septal geometry and myofiber mechanics in the three ventricular walls. The interesting feature of the CircAdapt model is its combined adaptation of the heart and vessels over a relatively long period resulting in self-structuring of the circulation as a system where a steady-state solution is obtained [74,75]. This feature makes the model a potential tool for clinical application with the aim of predicting the evolution of a diseased status and the effects of an interventional procedure [1,71,72,76,77], although there is a lack of suitable models for VAD support. Despite its limitations, the CircAdapt model is considered easy to use, requires relatively low computational time and allows realistic simulations of the circulation with boundary conditions suitable for more complicated models based on finite element analysis. HeMoLab (Haemodynamics Modelling Laboratory) is an integrated computational environment for the modeling of the cardiovascular system. It is an effective research tool and a virtual simulation laboratory [78,79,80]. HeMoLab consists of a combination of models, which can be coupled locally and globally in order to obtain the systemic response of the cardiovascular system: the so-called 3-D, 1-D and 0-D models. The propagation of the arterial pulse is represented with a 1-D and 0-D model, which describes the behavior of the flow rate, mean pressure and cross-sectional area as a function of time. HeMoLab is a suitable environment for the simulation of the effects of aging, vasodilatation, vasoconstriction, rest and exercise and calculation of characteristic impedance of the arterial network. Although attractive, the software remains confined to a research environment at present. Harvi is an interactive simulation textbook of cardiovascular physiology and hemodynamics based on a previously described electrical circuit [81,82,83] where ventricular and atrial contraction are represented by a modified time-varying elastance approach [5]. SimVascular is another attractive software package providing tools for medical image data segmentation and patient-specific blood flow simulation and analysis [84,85]. The SimVascular Supercomputing Gateway gives access to high-performance computing (HPC) clusters to run simulations using SimVascular solvers for computational fluid dynamics (CFD). Simulation software such as CARDIOSIM^©^ and SimVascular address different aspects of patient-specific modeling. CARDIOSIM^©^ is predominantly based on pressure-volume analysis of the interactions between mechanical circulatory assist devices (pulsatile and continuous flow VADs, IABP, TAL, BiVAD, ECMO), mechanical ventilatory support and the cardiovascular system. The coupling with lumped-parameter models gives detailed hemodynamic and energetic analysis in real time. SimVascular gives the opportunity to develop 3D reconstruction from CT-scan images for fluid dynamic analysis in terms of blood flow, pressure and shear stress based on specific boundary conditions. Coupling of 3-D to 1-D or 0-D models is used to reduce computational time with great effect. Both CARDIOSIM^©^ and SimVascular achieve the desired outcome despite their own limitations. All these software choices have attractive features and great potential. CARDIOSIM^©^ may be a more suitable choice in view of its features, particularly with reference to the effects of mechanical circulatory support on the cardiovascular system.

Lumped-parameter models assume a uniform distribution of pressure, volume and flow within any specific compartment at any instant in time, while higher dimensional models recognize the variation in space of these parameters. Lumped-parameter models consist of simultaneous ordinary differential equations complemented by an algebraic balance equation. They are suitable for examination of global distribution of pressure, flow and volume over a range of physiological conditions with the inclusion of the interaction between modeled components. Higher dimensional models consist of partial differential equations complemented by balance equations. One-dimensional models represent wave transmission effects within the vascular system, but 3-D numerical solutions are required for complex flow patterns, with analytical solutions obtained only for the simplest geometry [44]. A single-branch multiple-compartment model of the vascular system is suitable for the evaluation of short-term VAD support compared to an overly detailed vessel branch model where parameter setting becomes quite difficult. Higher level lumped-parameter modeling is required to address the interaction between the circulation and other systems, but a compromise between complexity and ability to set the required parameters is needed to personalize an integrated lumped model for a patient-specific approach. CARDIOSIM^©^ does address the system interaction with its modular approach and assembly of models with varying degrees of complexity, although 0-D and 1-D coupling may be required for the evaluation of long-term VAD support.

The accuracy and reliability of CARDIOSIM^©^ software have been confirmed by statistical analysis with Student’s t-test and the K Cohen index [86]. CARDIOSIM^©^ has the potential to deliver reliable simulations for a more quantitative and critical evaluation of device treatment and optimization in advanced heart failure. The clinician remains the ultimate decision-maker, who relies on an additional tool that may reduce unnecessary guesswork and perhaps give reassurance.

A simulation setting may well add a quantitative approach to help the decision-making process, generate more critical thinking and possibly reduce uncertainty. The development of an integrated description of the cardiovascular system based on lumped-parameter models and modified time-varying elastance with pressure–volume analysis of ventricular function seems a feasible and suitable approach generating an accurate quantitative analysis in real time. The challenge remains the ability to predict outcome over a longer period.

## Figures and Tables

**Table 1:** Evolution over time of the ventricle, atrium and septum models.

First Version	Second Version	Version 7.3.2
Ventricular filling and ejection phases are modeled separately to reproduce the behavior of the left and right ventricles. The time-varying elastance theory is used to reproduce the contraction and ejection phases [10,40].	Ventricular filling and ejection phases are modeled separately to reproduce the behavior of the left and right ventricles. The time-varying elastance theory is used to reproduce the contraction and ejection phases.	Ventricular filling and ejection phases are modeled separately to reproduce the behavior of the left and right ventricles. The time-varying elastance theory is used to reproduce the contraction and ejection phases.
A linear capacity assuming a constant value is used to reproduce the behavior of both the right and left atria [10,40]. Only a passive phase of the atria (left and right) can be reproduced using a compliance with a constant value.	A linear capacity assuming a constant value is used to reproduce the behavior of both the right and left atria [10,40]. Only a passive phase of the atria (left and right) can be reproduced using a compliance with a constant value.	A linear capacity assuming a constant value is used to reproduce the behavior of both the right and left atria [10,40]. Only a passive phase of the atria (left and right) can be reproduced using a compliance with a constant value.
--------	--------	Both ventricles are modeled according to the time-varying elastance concept [13,19,41,42].
--------	--------	Both atria are modeled according to the time-varying elastance concept [13,19,41,42].
--------	--------	The time-varying elastance theory is used to reproduce the septal activity [13,19,41,42].
		The time-varying interventricular and interatrial septum is modeled [13].

**Table 4:** Evolution over time of numerical models of mechanical circulatory assist devices.

First Version	Second Version	Version 7.3.2
Numerical model of pneumatic left ventricular assist device (LVAD) [40].	Numerical model of pneumatic left ventricular assist device (LVAD).	Numerical model of pneumatic left ventricular assist device (LVAD).
--------	Numerical model of pneumatic right ventricular assist device (RVAD).	Numerical model of pneumatic right ventricular assist device (RVAD).
--------	Numerical model of pneumatic biventricular assist device (BVAD) [56].	Numerical model of pneumatic biventricular assist device (BVAD).
---------	Numerical model of pneumatic total artificial heart (TAH).	Numerical model of pneumatic total artificial heart (TAH).
---------	First numerical model of intra-aortic balloon pump (IABP) [57,58].	First numerical model of intra-aortic balloon pump (IABP).
---------	---------	Numerical model of intra-arterial axial flow blood pump (Hemopump^®^ HP31) connected to the cardiovascular network as LVAD and/or RVAD [59,60].
---------	---------	Numerical model of pulsatile LVAD blood flow (PUCA pump) [61].
---------	---------	Numerical model of biventricular pacemaker (BiV) [43].
---------	---------	Numerical model of thoracic artificial lung (TAL) [62].
---------	---------	Numerical model of centrifugal blood pump connected to the cardiovascular network as LVAD and/or RVAD [62].
---------	---------	Second numerical model of intra-aortic balloon pump (IABP) [63].
---------	---------	Numerical model of Impella (LVAD)*.
---------	---------	Numerical model of extra-corporeal membrane oxygenation [51].
---------	---------	Numerical model of ECMO coupled with the first numerical model of intra-aortic balloon pump (IABP) *.
---------	---------	Numerical model of ECMO coupled with Impella (LVAD) *.

* Presented at international conference but not published in peer-reviewed journal.

## Data Availability

No new data were created or analyzed in this study. Data sharing is not applicable to this article.
